# Laparoscopic vs. laparotomy tubal recanalization for fertility restoration after tubal sterilization: a retrospective analysis

**DOI:** 10.3389/fsurg.2026.1774235

**Published:** 2026-03-23

**Authors:** Yan Zhang, Yuan Lin, Xiujuan Chen, Xiaoqing Liu, Xiumei Xiong

**Affiliations:** Department of Obstetrics and Gynecology, Fujian Maternity and Child Health Hospital, College of Clinical Medicine for Obstetrics & Gynecology and Pediatrics, Fujian Medical University, Fuzhou, China

**Keywords:** laparoscopy, laparotomy, minimally invasive surgery, pregnancy outcome, tubal recanalization

## Abstract

**Objective:**

To compare the therapeutic effects of laparoscopic recanalization and laparotomy recanalization on tubal patency restoration after tubal sterilization.

**Methods:**

A retrospective analysis was conducted on the clinical data of 122 patients who underwent tubal sterilization surgery at one tertiary grade A hospital from May 2013 to May 2023 were retrospectively analyzed.. The patients were divided into the laparoscopic group (*n* = 61) and the laparotomy group (*n* = 61) based on the surgical approach. The recanalization status, clinical indicators, and postoperative pregnancy outcomes were observed and compared between the two groups.

**Results:**

The laparoscopic group had a higher recanalization rate, shorter postoperative ambulation, first flatus recovery, and hospital stay times, lower 12-hour pain scores, and less intraoperative blood loss (all *P* < 0.05). It also showed a higher intrauterine pregnancy rate and lower missed abortion and infertility rates (*P* < 0.05), with no significant difference in ectopic pregnancy rates (*P* > 0.05). Overall pregnancy rate was 66.39%, significantly associated with age (80.0% for ≤35 vs. 50.8% for >35 years, *P* = 0.001) and post-anastomosis tubal length (0% for <5 cm, 58.62% for 5–8 cm, 100% for >8 cm, *P* < 0.001). Univariate and multivariate regression identified age at recanalization and reconstructed tubal length as independent predictors (*P* < 0.05); other factors showed no significant associations (*P* > 0.05). The ROC curve for tubal length had an AUC of 0.8808(95%CI: 0.8227-0.9389), indicating reliable predictive value.

**Conclusion:**

Laparoscopic recanalization for patients after tubal sterilization is associated with less intraoperative blood loss, faster postoperative recovery, and higher recanalization and intrauterine pregnancy rates compared with open abdominal recanalization. Age and reconstructed tubal length are critical predictors of pregnancy success, highlighting their importance in preoperative assessment and surgical planning.

## Introduction

Tubal ligation, a common clinical contraceptive procedure for women, achieves sterility by severing or ligating the fallopian tubes to prevent sperm-egg union (1). With the adjustment of China's fertility policies (e.g., the two-child and three-child policies) and the increasing trend of remarriage after divorce, a growing number of women who underwent tubal ligation are now seeking to restore their fertility (2, 3). While tubal ligation is a reliable contraceptive method, reproductive reactivation often requires recanalization surgery to restore tubal patency (4, 5). Currently, two primary surgical approaches are used: laparotomy and laparoscopic recanalization. However, the literature remains inconclusive regarding the impact of these approaches on the rate of subsequent pregnancy.

Emerging evidence suggests that tubal recanalization can achieve pregnancy rates of 80%–90%, but surgical techniques may influence outcomes significantly (6). Traditional laparotomy, despite its efficacy, is associated with lengthy operation times, slow recovery, and suboptimal pregnancy rates, failing to meet patient expectations (2). In recent years, laparoscopic recanalization has gained traction due to advancements in medical technology, demonstrating promising effects on reproductive outcomes (7). Microsurgical techniques in recanalization have been shown to enhance fertility potential while reducing the risk of ectopic pregnancy. The mechanistic and technical disparities between laparoscopic and open surgeries may underlie differences in repregnancy rates, yet existing studies have not reached a consensus on this disparity (8, 9). Clarifying these effects is crucial for guiding clinical decision-making and addressing patient needs effectively.

This study aims to comprehensively compare the impact of laparoscopic and laparotomy recanalization on the repregnancy rate after tubal ligation. By analyzing surgical outcomes, recovery metrics, complication rates, and pregnancy outcomes in a retrospective comparative cohort, we seek to provide evidence-based recommendations for clinical practice. Specifically, we aim to determine whether laparoscopic recanalization offers superior reproductive outcomes compared to laparotomy, while evaluating factors such as operation time, postoperative pain, postoperative recovery time, and long-term fertility. This research intends to bridge the current evidence gap and assist clinicians in tailoring treatment strategies to optimize patients' chances of successful re-pregnancy.

## Materials and methods

### General information

Clinical data of 122 patients who underwent tubal sterilization surgery at Fujian Maternity and Child Health Hospital from May 2013 to May 2023 were retrospectively analyzed. Patients were categorized into two groups based on different surgical methods: 61 patients who underwent open tubal recanalization were assigned to the control group, and another 61 patients who received laparoscopic tubal recanalization were allocated to the study group. All operating surgeons were chief physicians with professional training and strictly followed standardized surgical procedures. Our study adhered to the STROBE guidelines. During the research, we strictly defined the inclusion and exclusion criteria for research subjects to ensure the representativeness and comparability of the samples. Data collectors were trained, and standardized and objective data collection forms were used for statistics. Special personnel were assigned to supervise the data collection and analysis processes and review the research results to reduce selection bias in the data.

The inclusion criteria were as follows: patients were no more than 45 years old and had undergone tubal sterilization; patients had regular menstrual cycles; ovarian function was normal, and there was no evidence of male infertility; and patients had complete clinical data. The exclusion criteria included: the presence of gynecological diseases causing infertility (such as endometriosis, space—occupying lesions of the uterus or ovaries, etc.) detected during surgery; patients with severe gynecological diseases (such as pelvic adhesions, etc.); and patients with incomplete general data. This study was approved by the Medical Ethics Committee of Fujian Maternity and Child Health Hospital (Grant No. 2024KY261). All procedures were conducted in accordance with the ethical standards of the Declaration of Helsinki (revised in 2013). Verbal informed consent was obtained from all participants through telephone follow - up.

### Surgical procedures for tubal recanalization

In the study group, patients underwent laparoscopic tubal recanalization under general anesthesia with tracheal intubation in the lithotomy position. A 10 - mm umbilical incision was made to establish a carbon dioxide pneumoperitoneum (10–12 mmHg) for laparoscope insertion. Then, three incisions, each 5 mm in length, were made on both sides of the lower abdomen. Methylene blue solution was injected via the cervix to locate tubal obstruction. 0.9% sodium chloride solution was injected subserosally at the ligated fallopian tube segment to form a hydrodissection plane, exposing the tubal core after serosa incision. Laparoscopic micro—scissors and vascular forceps dissected tubal scar to normal tissue, and the obstructed segment was excised. Patency testing was done: an anesthesia catheter was inserted through the fimbria to check distal patency (left in place), and diluted methylene blue via the hysterosalpingography tube checked proximal patency. The distal anesthesia catheter was inserted into the proximal lumen. 5–0 absorbable sutures interrupted the tubal core muscular layer at 4, 8, and 12 o'clock (4–6 stitches), then the serosal layer. The catheter was removed, and methylene blue injection confirmed smooth fimbrial overflow. Bipolar electrocoagulation (≈0.5s per session, repeated) controlled bleeding, and the operation ended after no abnormalities. In the control group, open tubal recanalization was done under general anesthesia with tracheal intubation (laryngeal mask). A ∼5 cm transverse lower abdominal pubic incision was made to enter the abdominal cavity. Tissue clamps fixed fallopian tubes at original ligation nodules. 0.9% sodium chloride solution was injected subserosally at original ligation scar ends, dissecting and dilating tubal layers. After serosa opening, mesosalpinx was separated (keeping fimbria infundibulum drooping). After removing occluded tube and scar, epidural anesthesia catheters were inserted into proximal and distal ends, with 0.9% sodium chloride injection to check patency. A microscopic anastomosis device fixed the fallopian tube broken end, and 5–0 absorbable sutures interrupted the tubal core broken end at 4, 8, and 12 o'clock (more stitches if needed). After hemostasis, 5–0 absorbable sutures continuously sutured the serosal layer, then 0.9% sodium chloride was injected and the catheter removed; the contralateral tube was treated similarly. For both groups, sodium hyaluronate was placed in the pelvis post—surgery, antibiotics were used for 48 h, contraception was required in the surgery month, and hysterosalpingography was repeated when menstruation was clean for 3–7 days post—surgery.

### Observation indicators and efficacy evaluation criteria

Parameters compared between groups included: perioperative indicators (operation duration, intraoperative blood loss, time to first postoperative flatus, time to ambulation, length of hospital stay, and 12-hour postoperative pain score). Pain was assessed using the visual analogue scale (VAS; 0–10, higher scores indicating more severe pain) (10). Postoperative complications included infection, bleeding, and exacerbated lower abdominal pain. Treatment efficacy was categorized as clinical cure (bilateral tubal patency with no/mildly resolvable resistance, no reflux, no abdominal pain, or ultrasound-confirmed intrauterine pregnancy), effective (partial patency with initially marked but subsequently reduced resistance, mild reflux and/or mild abdominal pain, or ultrasound-confirmed ectopic pregnancy), or ineffective (persistent occlusion with high injection resistance, total injected volume <8 mL, obvious reflux, and severe abdominal pain). The total effective rate was calculated as [(clinical cure+effective cases)/total cases] × 100%. Postoperative tubal patency was assessed by follow-up evaluation (including hysterosalpingography as described above) and classified as bilateral patency, unilateral patency, or bilateral occlusion. Pregnancy outcomes were limited to spontaneous conceptions (excluding assisted reproductive technology), with a 2-year postoperative follow-up to record intrauterine pregnancy, missed abortion, ectopic pregnancy, infertility, and live birth (delivery of at least one live-born infant).

### Statistical analysis

All data in this study were processed using SPSS 22.0 statistical software. Measurement data were expressed as $\bar{x}$ ±s and analyzed by *t*-test; counting data were expressed as percentages (%) and analyzed by *χ*^2^ test. A statistically significant difference between groups was considered when *P* < 0.05.

## Results

### Comparison of demographic characteristics between the Two groups of participants

In this study, the baseline characteristics of patients in the two groups were comparable, with no statistically significant differences (Table 1). In the laparoscopic group, the age at recanalization ranged from 28 to 43 years, with a mean age of (35.16 ± 3.92) years; in the laparotomy group, the age ranged from 28 to 44 years, with a mean age of (35.41 ± 3.78) years. There were no statistically significant differences between the two groups in terms of mean ligation duration, parity, or menstrual cycle. There was no known male infertility in any case. From the total patient group, 1 patient had type 2 diabetes mellitus, 1 had hypertension, and 2 had thyroid disorders. Additionally, no statistically significant differences were observed between the two groups in terms of ligation methods, ligation sites, or tubal length after recanalization. These results indicate that the two groups were comparable, and thus, baseline characteristics would not affect the study outcomes.

**Table 1:** Demographic and disease history characteristics of participants [ $\bar{x}$ ±s, n(%)].

Characteristic	Laparoscopy	Laparotomy	t/*χ*^2^	*P*-value
Cases (no.)	61	61		
Age at Sterilisation (years)	27.44 ± 3.42	26.87 ± 4.19	0.828	0.409
Age at Recanalisation (years)	35.16 ± 3.92	35.41 ± 3.78	−0.353	0.725
Recanalisation age group (years)			0.296	0.586
≤35	34 (55.74%)	31 (50.82%)		
>35	27 (44.26%)	30 (49.18%)		
Sterilisation-Recanalisation Interval (years)			1.836	0.399
<4	6 (9.84%)	5 (8.20%)		
4∼6	19 (31.15%)	13 (21.31%)		
>6	36 (59.02%)	43 (70.49%)		
Parity(no.)			2.033	0.154
≤2	61 (100%)	59 (96.72%)		
≥3	0 (0%)	2 (3.28%)		
Regularity of cycle			0.568	0.753
Always regular	58 (95.08%)	56 (91.80%)		
Sometimes regular	2 (3.28%)	3 (4.92%)		
Irregular	1 (1.64%)	2 (3.28%)		
Disease history			4.000	0.135
Diabetes mellitus	0 (0%)	1 (1.64%)		
Hypertension	1 (1.64%)	0 (0%)		
Hypothyroidism	0 (0%)	2 (3.28%)		
Method of ligation			0.307	0.580
Folding, ligation and amputation	35 (57.38%)	38 (62.30%)		
Extraction and embedding	26 (42.62%)	23 (37.70%)		
Site of ligation			0.067	0.967
Bilateral isthmo-isthmic	35 (57.38%)	34 (55.74%)		
Bilateral isthmoampullary	9 (14.75%)	10 (16.39%)		
Bilateral ampulloampullary	17 (27.87%)	17 (27.87%)		
Length of reconstructed tube (cm)			3.470	0.176
>8	28 (45.90%)	19 (31.15%)		
5∼8	27 (44.26%)	31 (50.82%)		
<5	6(9.84%)	11(18.03%)		

### Comparison of clinical indicators between the two groups

As shown in Table 2, the clinical indicators of the two groups were compared. Although the operation time in the laparoscopic group was slightly longer than that in the laparotomy group, the difference was not statistically significant (*P* = 0.528). However, the time to postoperative ambulation, time to first flatus recovery, and length of hospital stay in the laparoscopic group were all shorter than those in the laparotomy group, with the postoperative hospital stay showing a statistically significant difference (*P* < 0.05). Additionally, the 12-hour postoperative pain score in the laparoscopic group was significantly lower than that in the control group (*P* < 0.001), and the intraoperative blood loss was significantly less (*P* < 0.001). No complications occurred in any patient in the laparoscopy group, while 2 patients in the laparotomy group developed wound site infection and 1 patient experienced aggravated abdominal pain.

**Table 2:** Comparison of clinical indicators between two groups（ $\bar{x}$ ±s）.

Clinical indicators	Laparoscopy	Laparotomy	t/χ^2^	*P*-value
Cases (no.)	61	61		
Operative time (min)	138.44 ± 24.54	134.92 ± 35.96	0.632	0.528
Intraoperative blood loss (mL)	9.30 ± 5.44	19.48 ± 18.04	−4.220	<0.001
Postoperative ambulation time (h)	16.80 ± 3.64	17.41 ± 3.93	−0.885	0.378
Time to first flatus recovery (h)	23.00 ± 4.83	24.26 ± 6.30	−1.242	0.216
Days of discharge after surgery (d)	4.64 ± 1.28	5.25 ± 1.62	−2.296	0.023
Length of hospital stay (d)	8.31 ± 1.96	8.66 ± 3.37	−0.689	0.492
Postoperative 12-h pain score (points)	2.62 ± 1.24	4.97 ± 1.53	−9.306	<0.001
Postoperative complications				
Infection	0	2	3.000	0.083
Bleeding	0	0		
Aggravated abdominal pain	0	1		

### Follow-up results of tubal patency and pregnancy outcomes

**Figure 1:**
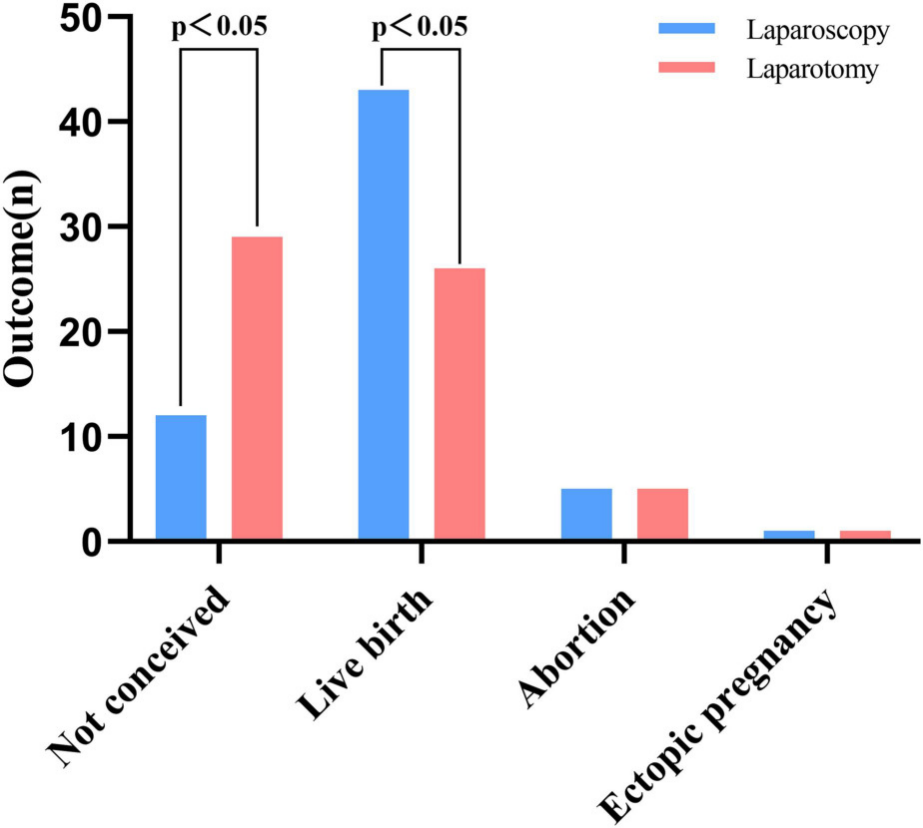
Comparison of pregnancy—related outcomes after laparoscopic and laparotomic tubal recanalization.

Although bilateral reconstruction was intended, bilateral tubal patency was not achieved in every patient. All patients were followed up to assess postoperative tubal patency (bilateral patency, unilateral patency, or bilateral occlusion; Table 3). In the laparoscopic group, 57 cases (93.44%) had bilateral patent fallopian tubes, and 3 cases (4.92%) had unilateral patency. In the laparotomy group, 55 cases (90.16%) achieved bilateral patency, while 5 cases (8.20%) showed unilateral patency. Both groups had 1 case of bilateral tubal occlusion. After two year of follow-up for patients who underwent tubal recanalization (Table 4), in the laparoscopic group, 43 cases (70.49%) had intrauterine pregnancy, 5 cases (8.20%) had miscarriages, 1 case (1.64%) had ectopic pregnancy, and 12 cases (19.67%) remained non-pregnant. In the laparotomy group, 26 cases (42.62%) had intrauterine pregnancy, 5 cases (8.20%) had miscarriages, 1 case (1.64%) had ectopic pregnancy, and 29 cases (47.54%) remained non-pregnant. As indicated in Figure 1, the pregnancy rate and live birth rate in the laparoscopic group were significantly higher than those in the laparotomy group, with statistically significant differences (*P* < 0.05).

**Table 3:** Comparison of recanalization rates between two groups [n(%)].

Tubal patency	Laparoscopy	Laparotomy	χ^2^	*P*-value
Cases (no.)	61	61	0.536	0.765
Bilateral patency	57 (93.44%)	55 (90.16%)		
Unilateral patency	3 (4.92%)	5 (8.20%)		
No. of patency	60 (98.36%)	60 (98.36%)		
Bilateral block	1 (1.64%)	1 (1.64%)		

**Table 4:** Comparison of pregnancy rates between two groups [n(%)].

Outcome	Laparoscopy	Laparotomy	χ^2^	*P*-value
Cases (no.)	61	61	10.617	0.001
Conceived	49 (80.33%)	32 (52.46%)		
Live birth	43 (70.49%)	26 (42.62%)		
Abortion	5 (8.20%)	5 (8.20%)		
Ectopic pregnancy	1 (1.64%)	1 (1.64%)		
Not conceived	12 (19.67%)	29 (47.54%)		

### Correlation analysis between pregnancy rate and influencing factors

A correlation analysis between the pregnancy rate and other conception-influencing indicators was conducted (Table 5). Among the 122 followed-up patients, 81 achieved pregnancy, including 69 with successful deliveries and 10 with spontaneous abortions (with unknown causes), resulting in an overall pregnancy rate of 66.39%. For the 41 non-pregnant patients who underwent salpingography, 39 showed complete patency, corresponding to a recanalization rate of 95.12%. Subgroup analysis demonstrated that among patients aged ≤35 years (*n* = 65), 52 achieved pregnancy, with a pregnancy rate of 80.0%, while among those aged >35 years (*n* = 57), 29 achieved pregnancy, with a pregnancy rate of 50.8%, and the difference between the two groups was statistically significant (*χ*^2^ = 11.544, *P* = 0.001). In terms of tubal length after anastomosis, 17 patients with a tubal length of less than 5 cm had a 0% pregnancy rate; 58 patients with a tubal length between 5 and 8 cm had 34 pregnancies, with a pregnancy rate of 58.62%; and 47 patients with a tubal length exceeding 8 cm all achieved pregnancy, with a 100% pregnancy rate, and the differences among these subgroups were statistically significant (*P* < 0.001).

**Table 5:** Conception rate in relation to various influencing factors [n(%)].

Variables	Conceived	Not conceived	χ^2^	*P*-value
Cases (no.)	81 (66.39%)	41 (33.61%)		
Recanalisation age group (years)			11.544	0.001
≤35	52 (80.00%)	13 (20.00%)		
>35	29 (50.88%)	28 (49.12%)		
Method of ligation			1.838	0.175
Folding, ligation and amputation	36 (73.47%)	13 (26.53%)		
Extraction and embedding	45 (61.64%)	28 (38.36%)		
Site of ligation			3.932	0.140
Bilateral isthmo-isthmic	50 (72.46%)	19 (27.54%)		
Bilateral isthmoampullary	13 (68.42%)	6 (31.58%)		
Bilateral ampulloampullary	18 (52.94%)	16 (47.06%)		
Sterilisation-Recanalisation Interval (years)			0.969	0.616
<4	8 (72.73%)	3 (27.27%)		
4∼6	23 (71.88%)	9 (28.12%)		
>6	50 (63.29%)	29 (36.71%)		
Tubal patency after recanalization			4.113	0.128
Bilateral patency	76 (67.86%)	36 (32.14%)		
Unilateral patency	5 (62.50%)	3 (37.50%)		
Bilateral block	0 (0.00%)	2 (100.00%)		
Length of reconstructed tube (cm)			58.946	<0.001
>8	47 (100.00%)	0 (0.00%)		
5∼8	34 (58.62%)	24 (41.38%)		
<5	0(0.00%)	17(100.00%)		

As shown in Table 5, intraoperative observations revealed that most cases with large ligation nodules and extensive serosal damage were associated with the folding, ligation and amputation technique (modified Pomeroy method), while those with small ligation nodules and minimal serosal damage were mostly from the extraction and embedding technique. Among the 49 cases that underwent the extraction and embedding technique, 36 achieved pregnancy, with a pregnancy rate of 73.47%; among the 73 cases that underwent the modified Pomeroy method, 45 achieved pregnancy, resulting in a pregnancy rate of 61.64%. In terms of tubal ligation sites: 69 cases with isthmus-isthmus ligation had 50 pregnancies, with the highest pregnancy rate of 72.46%; 19 cases with isthmus-ampulla ligation had 13 pregnancies, with a pregnancy rate of 68.42%; and 34 cases with ampulla ligation had 18 pregnancies, with a pregnancy rate of 52.94%. In this study, most patients with bilateral tubal patency after recanalization achieved pregnancy (67.86%), and most cases with unilateral tubal patency also successfully conceived (62.5%). It is generally believed that the duration of ligation may potentially affect tubal recanalization outcomes. However, in this study, no significant statistical correlation was observed between the pregnancy rate and the interval from ligation to anastomosis: among 11 cases with ligation duration <4 years, 8 achieved pregnancy (72.73%); among 32 cases with ligation duration of 4–6 years, 23 achieved pregnancy (71.88%); and among 79 cases with ligation duration >6 years, 50 achieved pregnancy (63.29%).

### Analysis of influencing factors on pregnancy rate

**Figure 2:**
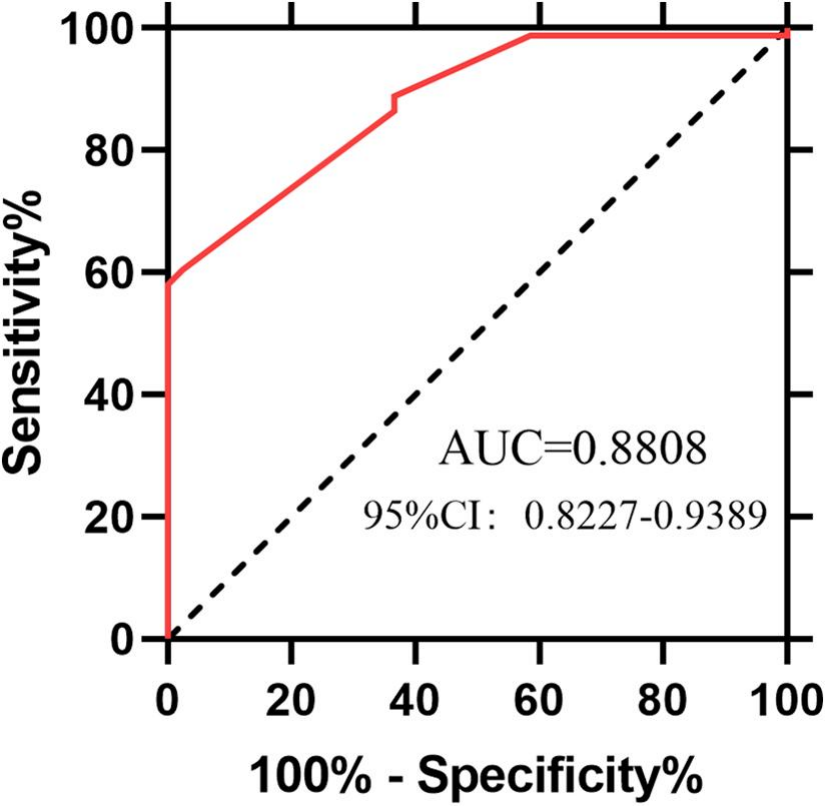
Receiver operating characteristic (ROC) curve for predicting pregnancy success based on the length of the reconstructed fallopian tube.

Univariate and multivariate regression analysis models were used to explore factors associated with the pregnancy rate (Table 6). In the univariate analysis, patient age at recanalization and the reconstructed tubal length after recanalization showed statistically significant trends in association with the pregnancy rate. Further multivariate regression analysis revealed that age at recanalization and reconstructed tubal length were independent significant predictors of the pregnancy rate. After multivariate adjustment, factors such as ligation method, interval between sterilization and recanalization, and tubal patency after recanalization were not significantly associated with the pregnancy rate (*P* > 0.05). A receiver operating characteristic (ROC) curve was constructed to evaluate the predictive value of tubal length for pregnancy outcomes (Figure 2). The area under the curve (AUC) was 0.8808, with a 95% confidence interval (CI) ranging from 0.8227 to 0.9389, suggesting that tubal length could serve as a relatively reliable indicator for predicting pregnancy outcomes after tubal recanalization. These findings highlight the critical roles of patient age and reconstructed tubal length in predicting pregnancy success following tubal recanalization.

**Table 6:** Factors associated with conception rate using univariate and multivariable analysis models.

		Univariate regression (*p* < 0.1)			Multivariate regression (*p* < 0.05)		
Factor	n	*β*	Standard error	*P* value	β	Standard error	*P*-value
Recanalisation age group (years)							
≤35	65	−0.291	0.082	0.001	−0.161	0.068	0.019
>35	57	-	-	-	-	-	-
Method of ligation							
Folding, ligation and amputation	49	−0.118	0.087	0.178	−0.082	0.066	0.212
Extraction and embedding	73	-	-	-	-	-	-
Site of ligation							
Bilateral isthmo-isthmic	69	−0.094	0.049	0.055	−0.033	0.037	0.378
Bilateral isthmoampullary	19	-	-	-	-	-	-
Bilateral ampulloampullary	34	-	-	-	-	-	-
Sterilisation-Recanalisation Interval (years)							
<4	11	−0.060	0.066	0.360	0.022	0.051	0.672
4∼6	32	-	-	-	-	-	-
>6	79	-	-	-	-	-	-
Tubal patency after recanalization							
Bilateral patency	112	−0.200	0.122	0.104	0.016	0.091	0.857
Unilateral patency	8	-	-	-	-	-	-
Bilateral block	2	-	-	-	-	-	-
Length of reconstructed tube (cm)							
>8	47	0.478	0.046	<0.001	0.449	0.047	<0.001
5∼8	58	-	-	-	-	-	-
<5	17	-	-	-	-	-	-

## Discussion

Tubal ligation, a procedure that severs or blocks the fallopian tubes to prevent the normal passage of ova, boasts a high contraceptive success rate with minimal physical damage to patients. Due to its safety, superior contraceptive efficacy compared to other daily contraceptive methods, and negligible impact on reproductive function, it is widely chosen by women with no desire for future childbearing. However, in recent years, with the implementation of the national two-child policy, some patients have sought to restore their fertility. Tubal ligation reversal surgery, which addresses this need, has thus become crucial for achieving subsequent pregnancies. The most common clinical approaches include traditional laparotomy and laparoscopic surgery. Although both methods have their respective advantages, there remains considerable clinical debate regarding their comparative efficacy (11–13).

This study analyzed the efficacy of two tubal recanalization approaches, showing the laparoscopic group had a higher recanalization rate—outperforming open recanalization, which is straightforward but less effective in difficult cases (e.g., inconsistent bilateral tubal diameters). Laparoscopy, aided by adjustable-magnification microscopy, expands the surgical field for precise operation, enhances accuracy to support tubal function recovery and prevent postoperative obstruction, and aligns with prior studies (14, 15) in using microsurgical techniques (e.g., complete scar resection, atraumatic suturing to avoid mucosal inversion) to boost patency. The laparoscopic group also had shorter postoperative ambulation, first flatus recovery, hospital stay, lower 12-hour pain scores, and less intraoperative blood loss (all *P* < 0.05), reflecting faster recovery and better comfort—benefits of clear visualization (reducing tissue damage/trauma) and a closed operative environment (lowering complication risk). Notably, laparoscopy had slightly longer operation times, attributed to higher technical demands for instrument manipulation, additional steps (e.g., pelvic exploration, tubal dissection), and delicate maneuvers (e.g., preserving anastomotic blood supply) to ensure efficacy.

The results indicated that the laparoscopic group had a higher intrauterine pregnancy rate—this rate was higher than those reported in previous studies (16–19)—as well as lower rates of missed abortion and infertility compared with the laparotomy group (*P* < 0.05). Ectopic pregnancy rates were low in both groups and did not differ significantly (*P* > 0.05), which is consistent with previous reports (20). These findings suggest that laparoscopic recanalization can improve the likelihood of intrauterine pregnancy while offering faster recovery. Preoperative assessment of gynecologic status and, when appropriate, hysterosalpingography to evaluate surgical difficulty may help optimize patient selection and counseling. Intraoperatively, careful preservation of mesosalpinx blood supply is essential for maintaining tubal function, and when feasible, less traumatic techniques should be prioritized to minimize patient morbidity.

This study found that the pregnancy rate after recanalization is associated with the patient's age at the time of the procedure, which is consistent with previous study (21). Patients under 35 years of age have advantages in physical condition and ovarian function, whereas the success rate of subsequent pregnancy decreases with increasing age (22, 23). This is likely related to the physiological decline in reproductive function associated with aging. Additionally, in patients with a sterilization duration exceeding 5 years, reduced corpus luteum function, along with atrophy and ciliary loss of the tubal epithelium near the uterine stump, may also lower the pregnancy rate. Notably, there is a close correlation between tubal length and post-recanalization pregnancy success. Excessively short fallopian tubes after laparoscopic recanalization can cause early entry of fertilized eggs into the uterine cavity; simultaneously, if the fimbrial end fails to reach the ovarian surface, it impairs fimbrial egg retrieval and tubal peristalsis, hindering conception. Therefore, in clinical practice, prior to laparoscopic recanalization, assessments of the patient's age, sterilization duration, and tubal length are essential to guide surgical planning. For patients unsuitable for surgery or those with predicted poor outcomes, thorough explanation and counseling should be provided.

Consistent with previous research (24), our study revealed a strong association between the pregnancy rate after tubal recanalization and the length of the anastomosed tube: the pregnancy rate was 0% in cases with a postoperative tubal length <5 cm, 77.14% in those with a length >5 cm, and as high as 100% in cases with a length >8 cm. Multivariate regression analysis identified age at recanalization and reconstructed tubal length as independent significant factors influencing the pregnancy rate. The ROC curve yielded an AUC of 0.8808, indicating that tubal length is a decisive factor for post-recanalization pregnancy, with a minimum length of >5 cm required to achieve conception.In the previous study, 98.95% had a final tubal length of more than 4 cm (25). In addition, the site of tubal ligation directly affects the outcome of recanalization. Consistent with previous studies (26), the isthmo-isthmic ligation site yielded the highest pregnancy rate in the present study. Isthmic ligation is considered the optimal site for reversal: despite its small lumen, the isthmus has a thick muscular layer, uniform caliber, shallow mucosal folds, and fewer cilia, facilitating clear anatomical alignment during anastomosis. In contrast, isthmo-ampullary ligation involves mismatched luminal calibers, leading to poor alignment; ampullary ligation, characterized by a large lumen, thin muscular layer, and abundant cilia, may impair ciliary motility, resulting in successful recanalization but poor pregnancy outcomes due to failed functional recovery. Additionally, no significant correlation was observed between the pregnancy rate and the duration of ligation. Importantly, although bilateral reconstruction was intended, a small subset of patients achieved unilateral rather than bilateral patency at follow-up, highlighting real-world heterogeneity that can influence fertility outcomes.

This study has several limitations that should be considered when interpreting the results. Firstly, although a total of 122 patients were included in the analysis, the sample size for subgroup analyses was substantially smaller. This limited subgroup size may have compromised statistical power, increasing the risk of type II errors—potentially leading to the failure to detect clinically meaningful differences that actually exist between the two surgical groups. Secondly, the retrospective design of this study inherently introduces unavoidable biases. Patient selection for laparoscopic vs. open tubal recanalization was not randomized, which may have introduced selection bias; unmeasured confounding factors—such as subtle tubal pathologies (e.g., mild tubal mucosal damage) not detected intraoperatively, which may have influenced the observed differences in postoperative outcomes. Thirdly, bilateral tubal patency was not achieved in every patient; pregnancy outcomes therefore reflect real-world heterogeneity in reconstruction success and may not be fully generalizable to settings where bilateral patency is consistently achieved. Fourthly, the 2-year follow-up duration may not have fully captured all relevant fertility and safety outcomes. Some potential pregnancies (e.g., those occurring beyond 2 year post-surgery) or late complications (e.g., delayed pelvic adhesions or tubal reobstruction) might have been missed, which could lead to an underestimation of the true pregnancy rate or an incomplete assessment of long-term surgical safety. Finally, the study population was recruited exclusively from a single regional setting (southern China), with data derived from a single tertiary hospital. This regional homogeneity means the results may lack generalizability to populations with different demographic characteristics (e.g., age distribution, reproductive history), surgical practice patterns (e.g., variations in surgeon experience or intraoperative techniques), or healthcare access—limiting the applicability of our findings to other geographic regions or clinical settings.

In summary, the success rate of tubal recanalization primarily depends on the conditions during anastomosis. Among these, tubal length is one of the most critical factors. Other favorable conditions for successful recanalization and subsequent pregnancy include ligation at the isthmic segment using the extraction-embedding method (which results in small scars, minimal serosal damage, and preserved lumen integrity), as well as non-tortuous fallopian tubes. While all patients undergoing recanalization urgently desire successful anastomosis, surgical outcomes depend not only on the surgeon's expertise. This underscores the importance of standardized procedures for gynecologists performing tubal sterilization: they must consider the potential need for future recanalization, maximize the preservation of tubal length, and preferentially choose isthmic ligation with the extraction-embedding technique. Such practices not only improve the quality of tubal sterilization but also provide optimal luminal conditions for potential subsequent recanalization.

## Conclusion

Tubal recanalization enables patients to conceive again after sterilization, and its success rate is determined by multiple factors. In addition to the surgeon's proficient microsurgical techniques, the success rate of tubal recanalization is significantly related to the length of the fallopian tube after recanalization and the ligation site, and has a certain correlation with the ligation method and the patient's age at the time of recanalization, but has no relationship with the time interval between ligation and anastomosis. Laparoscopy has the advantages of minimally invasive surgery, fewer complications, and a higher pregnancy rate. Therefore, laparoscopic recanalization may be considered a preferred option in appropriately selected patients, depending on local expertise and patient-specific characteristics.

## Data Availability

The original contributions presented in the study are included in the article/Supplementary Material, further inquiries can be directed to the corresponding author.
